# Effects of Si Solution on Stability of Early 3d Transition-Metal Tri-Aluminides, Al_3_T (T = Sc, Ti and V)

**DOI:** 10.1007/s11837-024-06834-6

**Published:** 2024-09-05

**Authors:** C. M. Fang, Z. P. Que, Z. Fan

**Affiliations:** Brunel Centre for Advanced Solidification Technology (BCAST), Brunel University London, Uxbridge, Middlesex UB8 3Ph UK

## Abstract

Addition of the early 3d transition metals results in formation of primary Al_3_T (T = Sc, Ti and V) phases in Al alloys during casting. The newly formed Al_3_T particles not only improve the mechanical performance of the products but also act as grain-refiners in the solidification processes. Meanwhile, experiments found impacts of impurities, such as Si, on the formation of the Al_3_T phases; the mechanism is not fully understood. We here investigate effects of Si solution on the stability and crystal chemistry of the Al_3_T phases using first-principles density-functional theory. The study has revealed a rich variety of effects of Si solution on the Al_3_T phases. Si solution stabilizes the D0_22_-Al_3_Ti structure so that it becomes the ground state, taking over the binary D0_23_ phase. Si solution in D0_22_-V occurs only at elevated temperature. Si solution has little impact on the Al_3_Sc phase relationship. The obtained information helps characterize the (Al, Si)_3_ T particles in Al products, understand their role in solidification and further design new Al alloys of desirable properties.

## Introduction

Addition of transition metals can improve the mechanical performance and corrosion resistance of Al alloys.^1,2^ The early 3d transition metals have relatively low mass densities (3.0 g/cm^3^ for Sc, 4.5 g/cm^3^ for Ti and 6.1 g/cm^3^ for V), which are comparable to that of Al (2.7 g/cm^3^).^1,3^ This unusual character benefits manufacturing light-metal alloys of relatively small weight/volume ratios. The added early 3d transition metals during casting react with Al, forming Al_3_T particles.^4–7^ The newly formed micro-/nano-scaled Al_3_T particles cause the improvement of the mechanic performance and chemical properties of the products for aerospace and automotive transport applications.^7–12^

Those native Al_3_T particles may act as potential grain refiners during solidification of the Al alloys as well.^13–17^ The small lattice mismatch between the cubic L1_2_-Al_3_Sc^8^ and α-Al^3^ means high nucleation potency of L1_2_-Al_3_Sc substrates. Al_3_Ti particles have been considered to act as a grain refiner separately^18,19^ or to work together with TiB_2_ particles in the widely used Al-nTi-B (*n* = 3, 5) master alloys.^20–22^ Native Al_3_V particles perform grain refinement in the Al(V) alloy.^16,17^ Moreover, the early 3d transition metal tri-aluminides may also be formed at the joints during welding, e.g., Al_3_Ti at Ti-containing metals/Al joints.^23^ Thus, it is vital to have a comprehensive understanding of the phase relations, crystal structure and physical properties for furthering development of Al alloys of desirable properties, particularly for the recycling Al scrap/waste parts which may contain various impurities.^24,25^

**Fig. 1:**
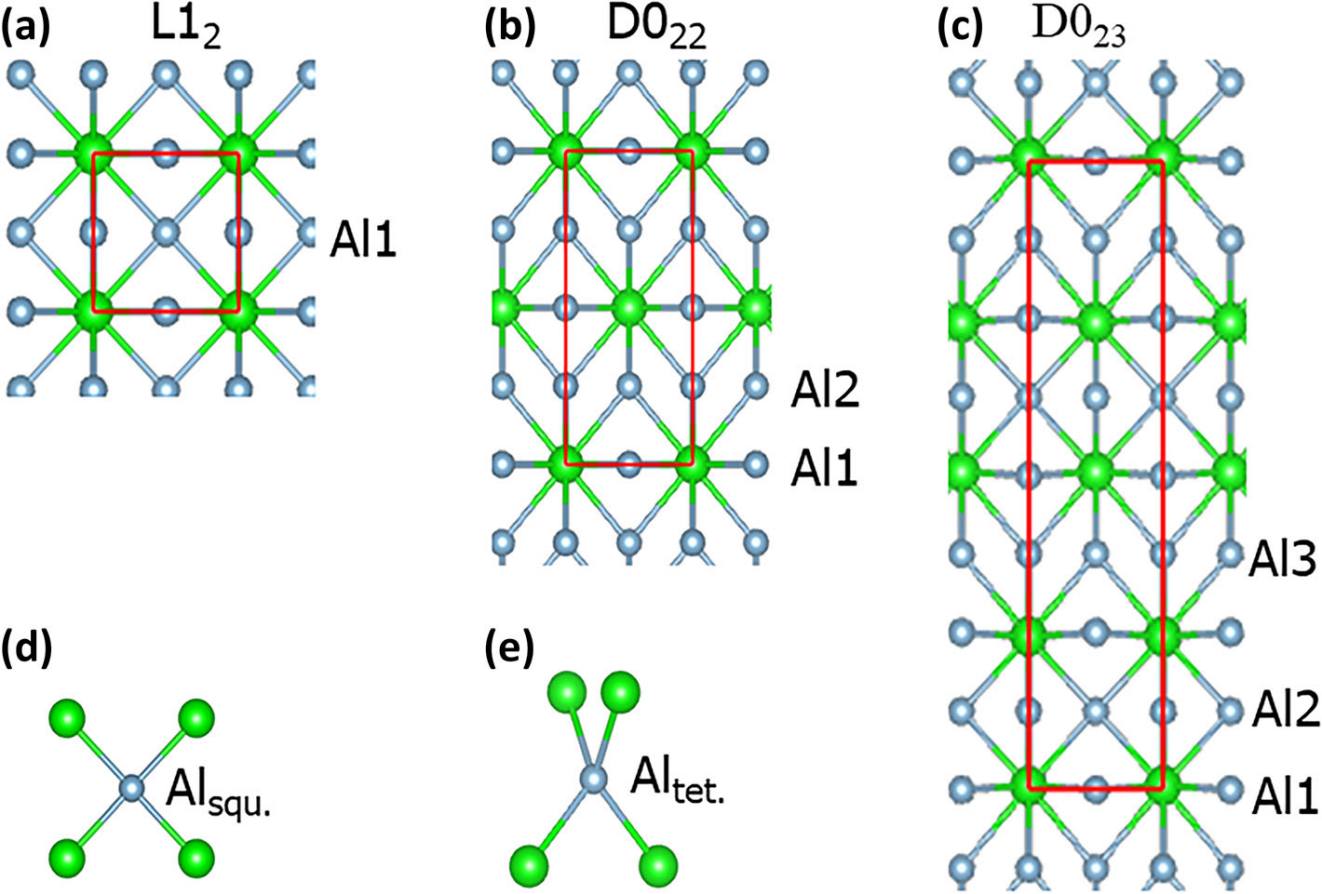
Schematic structures of the L1_2_- (a), D0_22_- (b) and D0_23_-TAl_3_ (c) projected along the [100] orientations and local coordination of Al atom in a square-planar (d) and tetrahedral (e) coordination by four T atoms. The red lines mean the *b*-axis (horizontal) and *c*-axis (vertical) in (a), (b) and (c). The *z*-component of the unit cell for D0_23_-TAl_3_ has been shifted by 1/8, which sets the Al atoms at the Wyckoff 4e site atoms at *z* = 0. The larger green spheres represent Ti and smaller silvery Al

There are the three most likely phases for the tri-aluminides: the cubic L1_2_- and the tetragonal D0_22_- and D0_23_-Al_3_T for T = Sc, Ti and V.^26,27^ The terms ‘Al_3_T’ and ‘TAl_3_’ are exchangeable in rest of the paper. Their structures are schematically drawn in Fig. 1.

In the cubic L1_2_-Al_3_T structure (Fig. 1a), each T atom has 12 Al nearest neighbours in cuboctahedral coordination. Each Al atom also has 12 neighbours (4 T and 8 Al) and is in square-planar coordination of T (Fig. 1d).

In the tetragonal D0_22_ phase, all atoms are still in the ideal positions, but the symmetry is broken (Fig. 1b). The T atoms are in distorted cuboctahedral coordination of Al. There are two types of Al coordination by T. One third of the Al atoms positioned in the same planes with T (with the coordinate component, *z* = 0.0 and 0.5 in Fig. 1b) are in square-planar coordination by T (Fig. 1d). The rest (with *z* = 0.25 and 0.75) are tetrahedrally coordinated by T (Fig. 1e).

In the D0_23_ phase, not only is the symmetry broken but also the atomic positions deviate from the ideal sites. The local coordination thus becomes distorted. All the T’s are still in distorted cuboctahedral coordination by Al. One third of the Al atoms (*z* = 0.375 and 0.875 in Fig. 1c) are in the distorted tetrahedral coordination by T, and the other two thirds are in distorted square-planar coordination with the Al being out of the T planes.

Overall, the L1_2_, D0_22_ and D0_23_ structures (Fig. 1) can be regarded as face-centred cubic (FCC) superstructures. The different coordination, broken symmetry and local structural distortion in the D0_22_ and D0_23_ phases would have an impact on their stability and content of Si solution at the Al sites.

The phase relationships of the early 3d transition metal tri-aluminides have been a topic of intensive study both experimentally^4–7,26^ and theoretically.^27–29^ It is generally agreed that the ground state Al_3_Sc has the cubic L1_2_-type structure (Fig. 1a)^4,8,10,28^ and Al_3_V the tetragonal D0_22_-type structure.^7,11,16,29^

There have been intensive discussions about the phase relations for Al_3_Ti as summarized in Ref. 27 The advanced first-principles investigations established that the D0_23_ phase is the ground state for Al_3_Ti.^27,30^ Meanwhile, the experiments produced a scattering of results. The thermodynamics study for the binary Al-Ti phase diagrams suggested phase transition between a high-temperature phase to a low-temperature one with variable transition temperatures.^5,31,32^ Structural characterization revealed formation of D0_22_-Al_3_Ti particles in manufactured Al products.^33–35^ Furthermore, the lattice parameters for the Al_3_Ti phases in different samples vary notably.^27,33–35^ The latter is also true for the observed DO_22_-Al_3_V samples.^35,36^

Impurities including Si exist inevitably in commercial Al metals.^37^ Si is often added to Al to obtain products of desirable properties for applications at extreme conditions, e.g., high temperatures.^1,2^ Hence, it is necessary to have a good understanding of Si solution in the early 3d transition-metal tri-aluminides and the corresponding effects on their stability and structural properties.

There have been efforts on Si solution in the Al_3_T phases^38,39^ along with the intensive study on the binary phases.^4–8,10,26–30,40–42^ Recently, Dumbre *et al*. studied the impact of thermal treatments on formation of (L1_2_-)(Al,Si)_3_Sc in Al-Si-Sc alloys.^43^ Yao performed first-principles calculations on the elastic and electronic properties of L1_2_-(Al, Si)_3_Sc phase.^44^ Using first-principles density functional theory approach, Castillo-Sánchez *et al*. investigated Si substitution in the Al_3_(Zr, Ti) intermetallic compounds with the D0_22_- and D0_23_-type structures.^39^ Meanwhile, there is still a lack of comprehensive understanding of the Si solution in the early 3d transition metal tri-aluminides phases.

Here, we investigate Si solution in the Al_3_T (T = Sc, Ti and V) forms of L1_2_-, D0_22_- and D0_23_-type structures in a systematic way using a first-principles density-functional theory method. This study reveals that Si solution favors the Al sites in Ti square-planar coordination (Fig. 1d) and stabilizes the D0_22_-Al_3_Ti phase so that the D0_22_-(Al_1−*x*_Si_*x*_)_3_Ti phases become more stable than the binary ground state (D0_23_-Al_3_Ti) phase. The Si solution in D0_22_-Al_3_V occurs only at elevated temperatures. The obtained information here helps not only to understand the phase relationships and the rich variety of experimental results in the literature but also to design new Al alloys of desirable mechanical and chemical properties based on Al scrap/waste parts, which benefits improve our circular society in an environmentally friendly, sustainable way.

## Details of Computations

To assess the relative stability of the binary Al_3_T compounds, the energy difference between the investigated X-Al_3_T and the corresponding cubic L1_2_ phase is defined as:

1 $$ \Delta E_{{1}} = E\left( {{\text{X}} - {\text{Al}}_{{3}} {\text{T}}} \right) \, {-}E\left( {{\text{L1}}_{{2}} - {\text{Al}}_{{3}} {\text{T}}} \right) $$

Here, *E*(X-Al_3_T) and *E*(L1_2_-Al_3_T) represent the calculated total valence-electron energies for the related X phase and related L1_2_-Al_3_T phase, respectively.

For dilute solution of Si and the early 3d transition metals in the Al matrix, the solution energy is defined as:

2 $$ \Delta E\left( {{\text{M}}*} \right) \, = E\left( {{\text{Al}}_{{n - {1}}} {\text{M}}} \right) \, {-} \, \left[ {\left( {\left( {n - {1}} \right)/n} \right) \, \times E\left( {{\text{Al}}_{n} } \right) \, + E\left( {\text{M}} \right)} \right] $$

where *E*(Al_*n*−1_M), *E*(Al_*n*_), *E*(M) represent, respectively, the calculated energies for the substituted Al_*n*−1_M, Al_*n*_ and the elemental solid M. The calculated total valence-electron energy of the same supercell of Al_*n*_ is used for systematic error cancellation. The unit of the solution energy is eV per M.

For a ternary Si-doped (Al_1−*x*_Si_*x*_)_3_T phase, the formation energy regarding the elemental solids Si, Al and dilute solution of T in the Al matrix is defined as,

3 $$ \Delta E_{{\text{f}}} = E\left[ {\left( {{\text{Al}}_{{{1} - x}} {\text{Si}}_{x} } \right)_{{3}} {\text{T}}} \right)] \, {-} \, \left\{ {{3}\left( {{1} - x} \right)E\left( {{\text{Al}}} \right) \, + { 3}xE\left( {{\text{Si}}} \right) \, + E\left( {{\text{T}}*} \right)} \right\} $$

Here, *E*[(Al_1−*x*_Si_*x*_)_3_T)], *E*(Al), *E*(Si) and *E*(T*) represent the calculated total valence-electron energies for the (Al_1−*x*_Si_*x*_)_3_T phase, the elemental solids Al and Si, and the dilute solution of a 3d transition metal, T in the Al matrix (T*) in Eq. 2, respectively.

The unit for both Eqs. 1 and 3 is eV/f.u. (f.u. represents formula unit, (Al_1−*x*_Si_*x*_)_3_T). A negative Δ*E*_1_ value in Eq. 1 means that the X-Al_3_T is more stable than the L1_2_ phase. For Eq. 3, a negative value of the formation energy means favouring formation of the (Al_1−*x*_Si_*x*_)_3_T phase regarding the elemental solids, Al, Si and T*. At *T* = 0 K and *P* = 0 Pa, the calculated formation energy in Eqs. 2 and 3 is equal to the related reaction enthalpy when the zero-vibration contribution is ignored.

A 3*a*_0_ × 3*a*_0_ × 3*a*_0_ (*a*_0_ is the lattice parameter of the cubic Al unit cell) supercell which contains 108 Al atoms is employed to model the dilute solution of Si and an early 3d transition metal in the Al matrix. The 2*a*_0_ × 2*a*_0_ × 2*a*_0_ (3*a*_0_ × 3*a*_0_ × 3*a*_0_), 2*a*_0_ × 2*a*_0_ × 1*c*_0_ and 2*a*_0_ × 2*a*_0_ × 1*c*_0_ (*a*_0_ and *c*_0_ are the lattice parameters of the conventional cells for the corresponding structures) supercells are used for the cubic L1_2_-, tetragonal D0_22_- and D0_23_-Al_3_T phases, and they contain 32(108), 32 and 64 atoms, respectively. The large supercell 3*a*_0_ × 3*a*_0_ × 3*a*_0_ for the L1_2_ phase was utilized to justify the usage of the smaller supercells.

A plane-wave approach embedded in the first-principles package VASP (Vienna *Ab initio* Simulation Package)^45^ has been employed in the present study. The generalized gradient approximation (GGA)^46^ within the projector-augmented wave frame^47^ has been used for the correlation and exchange terms. This is because the GGA works better than the local density approximations (LDA) for transition metals and their compounds.^46,48,49^ Cut-off energies are reasonably high (*E*_CUT_/*E*_AUG_ = 400.0 eV/550.0 eV) compared with the default values of the atoms (*E*_MAX_/*E*_AUG_ = 245.3 eV/322.1 eV for Si, 180.2 eV/240.3 eV for Al, 116.1 eV/154.8 eV for Sc, 133.7 eV/178.3 eV for Ti, and 144.4 eV/192.5 eV for V, respectively). Dense *k*-meshes for the structural optimizations and total energy calculations are used, e.g., a 10 × 10 × 10 mesh with *k*-points ranging from 35(75) to 250(500) for the L1_2_- and (D0_22_-)Al_3_T supercells and a 10 × 10 × 6 mesh with *k*-points ranging from 45 to 300 for D0_23_-Al_3_T supercells, depending on the symmetry in the Brillouin zone based on the Monkhorst-Pack scheme.^50^ Test calculations for the cut-off energies and *k*-meshes provide that the present settings are reasonable with energy deviations being < 1 meV/atom.

## Results and Discussion

### Calculated Results for the Elemental Solids and Dilute Solutions

First, structural optimizations were performed for the elemental solids, α-Al with the face-centred cubic structure and Si with the diamond-type structure, as well as the early 3d transitions metals.^3,26^ Both Sc and Ti have simple hexagonal lattices, while V has a body-centred cubic structure.^3^ We also performed calculations for dilute solution of the early 3d transition metals in Al. The obtained results are listed in Table I. The available experimental data in the literature are included for comparison.

**Table I:** Calculated results (lattice parameters, formation energies and important interatomic distances) for the elemental solids, Al, Si, Sc, Ti and V, and the dilute solution of Si and the early 3d transition metals in the Al matrix

Element/ele.config.	Symmetry	Latt. para. (Å) and (Δ) (exp. data at 0 K^3^)	Interatomic distances (Å) and coordination type
Al [Ne]3s^2^ 3p^1^	Cubic Fm-3 m (no. 225)	*a* = 4.039(0.2%) (4.0325)	Al-Al: 2.86(×12) (cuboctahedral)
Si [Ne]3s^2^ 3p^2^	Cubic Fd-3 m (no. 227)	*a* = 5.468(+0.7%) (5.42982)	Si-Si: 2.37(×4) (tetrahedral)
Sc [Ar]4s^2^ 4p^0^ 3d^1^	Hexagonal P6_3_/mmc (no. 194)	*a* = 3.320(+0.5%) (3.3035) *c* = 5.157(− 1.9 %) (5.2552)	Sc-Sc: 3.21(×6), 3.32(×6) (distorted anticuboctahedral)
Ti [Ar]4s^2^ 4p^0^ 3d^2^	Hexagonal P6_3_/mmc (no. 194)	*a* = 2.929(− 0.5 %) (2.9451) *c* = 4.593(− 1.8 %) (4.6783)	Ti-Ti: 2.85(×6), 2.93(×6) (distorted anticuboctahedral)
V [Ar]4s^2^ 4p^0^ 3d^3^	BCC, Im-3 m (no. 229)	*a* = 2.978(− 1.5 %) (3.0223)	V-V: 2.58(×8) (cubic)
Dilute solution of impurities in Al matrix (see Eq. 2)			
Al_107_M	Local symmetry	Δ*E*(M*)	T-Al bonds (Å)
Sc*	O_h_	-1.04 eV/Sc	Sc - Al: 2.90(×12)
Ti*	O_h_	-1.18 eV/Ti	Ti - Al: 2.83(×12)
V*	O_h_	-0.57 eV/V	V - Al: 2.78(×12)
Si*	O_h_	+ 0.43 eV/Si	Si -Al: 2.84(×12)

The solution energy, Δ*E*(M*) is obtained according to Eq. 2. The electronic configurations for the elements are listed in which the element labels in brackets represents the core electrons. Δ = (*d*_calc_ − *d*_exp_)/*d*_exp_ × 100 represents the deviations of the computed lattice parameters (*d*_calc_) from the corresponding experimental values extended to 0 K (*d*_exp_) in Ref. 3 in the parentheses. BCC represents body-centred cubic.

The calculations reproduced the experimental values well for the simple s-p elements, Al and Si with deviations within 1%. Meanwhile, for the early 3d transition metals, the calculated lattice parameters deviate somewhat more, 1.5% for the cubic V and about 1.8%/1.9% for the *c*-axis of the hexagonal lattices for Sc/Ti, respectively, from the experimental values in the literature.^3^ The calculated local Al-T bond lengths in the dilute solutions decrease with increasing valence electrons in the order, Sc, Ti and V, which is in line with the atomic radii in the pure metals (Table I). The calculated solution energies for the early 3d transition metals in the Al matrix are negative, indicating solutions in Al matrix are favoured regarding the elemental solids. Meanwhile, Si solution in Al is not favoured with a notable formation energy of 0.43 eV/Si, which agrees with the previous calculations using the same approach.^37,48^ The calculated Si-Al interatomic distance is 2.84 Å, which is slightly shorter than that of the Al–Al bonds. This result indicates that Si prefers not dissolving in the Al lattice. Thus, the energy of bulk Si is used as a reference to assess the stability of the compounds in equilibrium.

### Binary Al_3_T Phases

Structural optimizations and total energy calculations were carried out for the binary Al_3_T phases. We also calculated the formation energies for the related D0_19_-Al_3_T structure.^26,27^ The calculations showed that the D0_19_-Al_3_T phases are notably less stable with the calculated energies being over 4.0 eV/f.u. higher than those of the corresponding L1_2_ phases. Thus, the results for the D0_19_-Al_3_T phases are not discussed in the present paper. The calculated results for the L1_2_-, D0_22_ and D0_23_ phases are listed in Table II. The experimental values available in the literature are included in parentheses for comparison.

**Table II:** Calculated results (lattice parameters, formation energies and intermetallic bonds) in the binary TAl_3_ (T = Sc, Ti, V) phases

Phase	Latt./Spacegroup	Latt. paras (Å), vol.(Å^3^/f.u.), Δ*E*_*1*_ (eV/f.u.)			Remarks
		Al_3_Sc	Al_3_Ti	Al_3_V	Ts are in Al cuboctahedral coordination
L1_2_	Cubic Pm-3 m (no. 221)	*a* = 4.103 (4.106^8^) (4.105^51^) *V* = 69.20 **Δ*E***_***1***_** = 0.0**	*a* = 3.977 (3.967^50^) (−) *V* = 62.91 Δ*E*_*1*_ = 0.0	*a* = 3.897 (−) (−) *V* = 59.19 Δ*E*_*1*_ = 0.0	Al is coordinated in T square-planar coordination (Fig. 1d)
D0_22_	Tetragonal I4/mmm (no. 139)	*a* = 4.021 (−) (−) (−) *c* = 8.805 (−) (−) (−) *V* = 71.17 Δ*E*_*1*_ = + 0.364	*a* = 3.841 (3.836 to 3.854^27^) (3.849^34^) *c* = 8.618 (8.584 to 8.612^27^*) (8.610^34^) *V* = 63.57 Δ*E*_*1*_ = − 0.108	*a* = 3.765 (3.722^36^) (3.779^52^) *c* = 8.307 (8.195^36^)* (−) (8.322^52^)* *V* = 58.86 **Δ*E***_***1***_** = − 0.541**	Two types of Al Al1 at 2b in T square-planar coordination (Fig.1d) Al2 at 4d in T tetrahedra coordination (Fig. 1e) *Experimental D0_22_-Al_3_Ti/Al_3_V samples prepared with different chemical compositions
D0_23_	Tetragonal I4/mmm (no. 139)	*a* = 4.039 (−) (−) *c* = 17.218 (−) (−) *V* = 70.23 Δ*E*_*1*_ = + 0.184	*a* = 3.895 (3.875 to 3.947^27^) *c* = 16.662 (16.679 to 16.926^27^*) *V* = 63.19 **Δ*E***_***1***_** = **− **0.131**	*a* = 3.806 (−) (−) *c* = 16.315 (−) (−) *V* = 59.08 Δ*E*_*1*_ = − 0.268	Three types of Al Al1 at 4e and Al2 at 4c are in distorted square-planar coordination by T with Al out of the Ti plane Al3 at 4d are in distorted tetrahedra coordination

The energy differences, Δ*E*_*1*_, are obtained via Eq. 1. The experimental lattice parameters available in the literature are included in parentheses together with the references. The most stable phases are marked in bold.

As shown in Table II, the present calculations produced the following results.

1. With increasing number of d electrons in the transition metal, L1_2_-Al_3_Sc, D0_23_-Al_3_Ti and D0_22_-Al_3_V are the ground-state phases. D0_23_-Al_3_Ti has a formation energy 0.02 eV/f.u. lower than its D0_22_ phase. This conclusion agrees with the previous works.^27–29^
2. The calculated lattice parameter of L1_2_-Al_3_Sc is close to the experimental values from different groups.^8,51^ Meanwhile, the experimental values for the ground-state D0_22_-Al_3_V differ notably from each other.^36,52^ The calculated lattice parameters are in between those of the available experimental data (Table II).
3. As summarized in the references Ref. 27 and 52, the experimental lattice parameters for both D0_22_- and D0_23_-Al_3_Ti phases in the literature vary in ranges within 2%. The calculated values are close to the experimental values.

Intrinsic defects in the Al_3_T phases have been investigated. There are high energy costs for Al substitutions of T in the ground state Al_3_T phases (L1_2_-Al_3_Sc, D0_23_-Al_3_Ti and D0_22_-Al_3_V). For example, it costs > 0.70 eV to replace one T by Al regarding T solution in the Al matrix. The calculations also produced high energy costs to produce Al vacancies, e.g., it costs 0.81 eV to create one Al vacancy in the L1_2_-Al_3_Ti phase. The calculations indicate unlikeliness for the intrinsic defects to occur. Therefore, we limit ourselves to Si dissolving at the Al sites in the compounds.

### Effects of Si Solution on Stability of the Al_3_T Phases

The study includes various configurations of Si solution at the Al sites in the Al_3_T phases. It showed that the Si atoms prefer uniform layer-resolved distributions in the structures. For example, for two Si doped at the Al1 sites in the D0_23_-Al_3_Ti supercell, there are four equal layers, *z* = 0.0, ¼, ½ and ¾ (Fig. 1c). The calculations showed that the formation energies for the configuration with two Si at the same layer (z = 0.0) and that with two Si at the nearby layers (*z* = 0.0 and 0.25) are, respectively, about 0.04 eV and 0.02 eV higher than that with the Si atoms uniformly distributed (Si1 at *z* = 0.0 and Si2 at *z* = 0.50). This helps us choose configurations of high stability. The results for Si solution at the Al sites in the Al_3_T phases are addressed separately in the following subsections.

#### Si Solution in Al_3_Sc

**Fig. 2:**
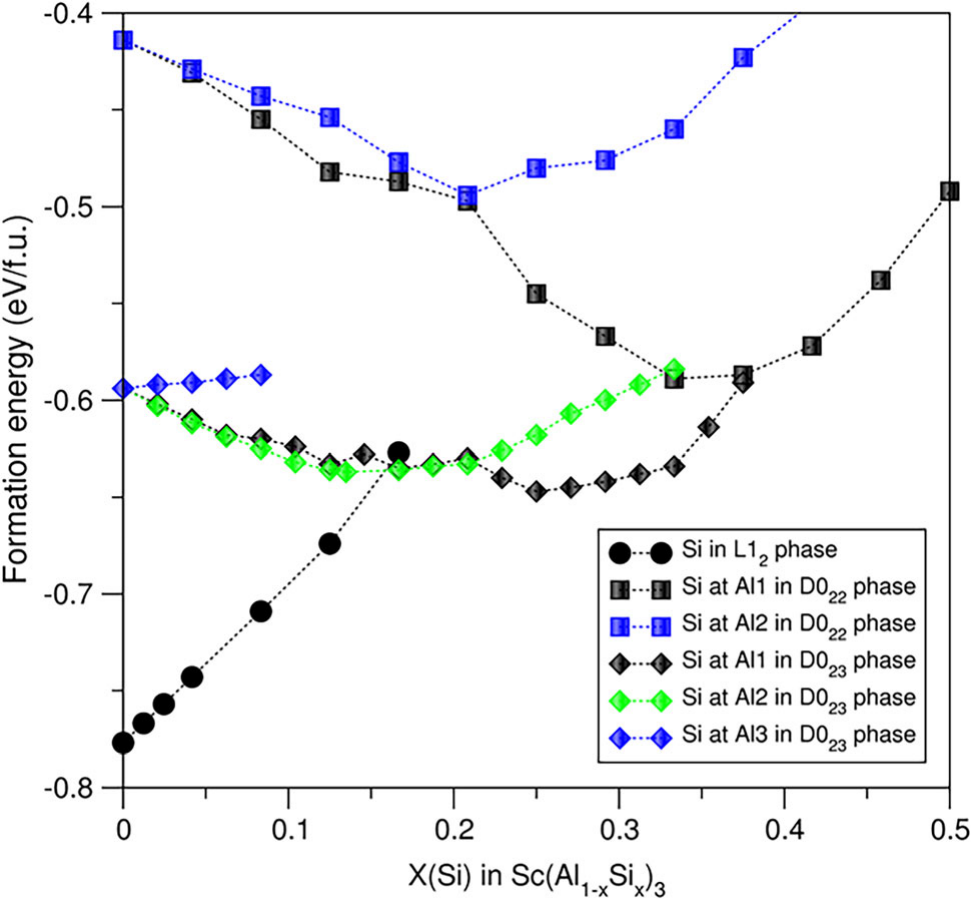
Dependences of the formation energies on Si content in the Al_3_Sc phases. Clearly, the binary L1_2_-Al_3_Sc is the most stable phase in the system

Figure 2 shows the calculated formation energies for the highly stable configurations with Si solution in the Al_3_Sc phases. Clearly, Si solution in L1_2_-Al_3_Sc costs a notable amount of energy. Doping one Si at the Al site costs 0.28 eV, indicating that it is highly unlikely to occur at low temperature. However, at high temperature, kinetic factor enables doping a moderate amount of Si at the Al sites, which agrees with experimental assumptions.^43^

Si solution at the Al sites in D0_22_-Al_3_Sc phases is favoured. With increasing Si content, the configurations become more stable. The most stable configuration is the full Si occupation of the Al1 site, which has the chemical formula D0_22_-Al_2_SiSc. This configuration gains a notable amount of energy (about 0.17 eV/f.u.). However, such an energy gain is not enough to make this phase more stable than L1_2_-Al_3_Sc. Addition of extra Si at the Al2 sites gradually reduces the stability of D0_22_-Al_2_SiSc.

For the D0_23_ phase, Si prefers the Al1 and Al2 sites where Al atoms are in distorted Ti square-planar coordination; meanwhile, Si solution at the Al3 sites with distorted Ti tetrahedral coordination costs moderate energy. The most stable configuration has the chemical formula D0_23_-(Al_0.708_Si_0.292_)_3_Sc. This formation energy is still much higher than the binary cubic L1_2_ phase, as shown in Fig. 2.

Overall, the calculations revealed preference of Si solution in D0_22_- and D0_23_-Al_3_Sc. The Si stabilization, however, is not strong enough to overtake the ground state L1_2_-Al_3_Sc phase. This study elucidates the experimental observations that this cubic phase exists and has been observed in the Al alloys. Moreover, the measured lattice parameters of L1_2_-Al_3_Sc in the Al alloys of different Si contents are close to each other,^8,43,51^ indicating minor Si content.

#### Si Solution Stabilizes D0_22_-Al_3_Ti

The calculations revealed that Si solution in L1_2_-Al_3_Ti is not favoured with an energy cost of 0.43 eV to replace one Al by Si. Such energy cost is close to that for doping one Si in bulk Al (Table I), indicating its occurrence is impossible.

**Fig. 3:**
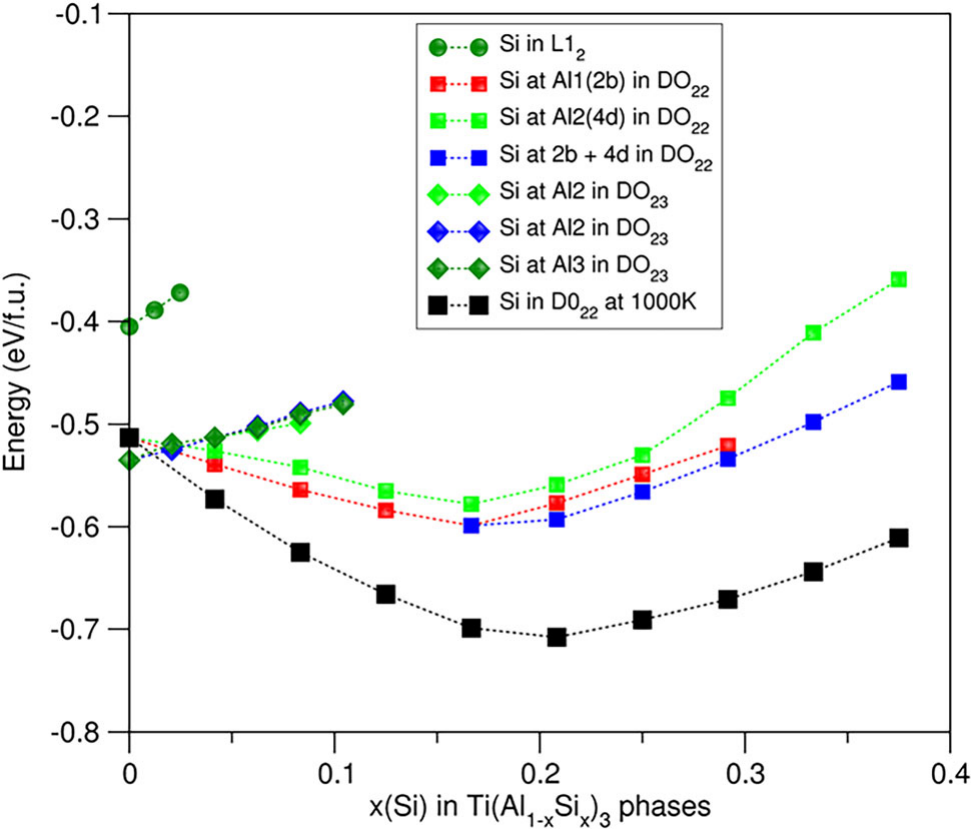
Relationships between the formation energies and Si content in the (Al_1−*x*_Si_*x*_)_3_Ti phases. Clearly, Si solution stabilizes the D0_22_ phase that it overtakes the D0_23_-Al_3_Ti phase as the ground state

Doping one Si atom at both Al1 and Al2 sites costs 0.16 eV and at the Al3 sites 0.28 eV in the D0_23_ phase, respectively. The Si doping at the Al1 sites is shown in Fig. 3. The formation energy increases almost linearly with the Si content in the D0_23_ phase.

Si solution at both Al1 and Al2 sites in D0_22_-Al_3_Ti is favoured. The formation energy for one Si at the Al1 sites is about 0.05 eV lower than that at the Al2 sites, indicating the former is preferred over the latter. The trends of relationships between the formation energies and Si content for Si solution at the Al1 and the Al2 sites are shown in Fig. 3. The formation energy decreases with increase of Si content and reach minima at *x*(Si) = 1/6 in (Al_1−*x*_Si_*x*_)_3_Ti. It then increases with further addition of Si. Thus, the most stable configurations have chemical composition D0_22_-(Al_0.833_Si_0.167_)_3_Ti with Si at Al1. Analysis showed that the most stable configuration of the composition, the Si atoms are distributed away from each other, in a uniform way. This Si content is notably lower than that in the reported D0_22_-(Al_2/3_Si_1/3_)_3_Ti with the Si full occupation of the 2b (Al1) sites, whose configuration has the minimum enthalpy in the Al_3_Ti-Si_3_Ti system in the recent publication (Fig. 12 in Ref. 39).

Figure 3 reveals that the formation energies for the configurations with > 2 at.% Si at the Al1 sites in the D0_22_ phase are lower than those of the ground state D0_23_-Al_3_Ti phase. This indicates that the Si-doped D0_22_ phase replaces the binary D0_23_-Al_3_Ti phase to be the ground state.

The partial occupation of Si at the Al sites indicates the important role of configurational entropy contributions in the stability at elevated temperature. The stability of the Si-doped D0_22_ phase at the casting temperature can be estimated via the Gibbs energy, Δ*G* = Δ*H* – *T* Δ*S*_*conf*_, where Δ*H* is the formation enthalpy being equal to Δ*E*_*f*_ at 0 K and 0 Pa, when the zero-point vibration contribution is ignored*, T* is the temperature, the configurational entropy, Δ*S*_*conf*_ = R ln*W* [R is the Boltzmann constant and *W* the number of configurations in the random model, considering kinetic factor over the moderate energy hierarchy (< 0.05 eV/f.u.) at such high temperature. The obtained values for the Gibbs energies at 1000 K are plotted in Fig. 3.

Figure 3 shows a shallow potential valley for the Gibbs energy on Si in the range *x* = 0.17 to 0.25 in the formula D0_22_-(Al_1−*x*_Si_*x*_)_3_Ti at the casting temperature with the minimum at *x* = 0.208. This indicates dependence of Si content in the obtained samples on the local chemical composition and thermal treatments.

In brief, Si solution is favoured in D0_22_-Al_3_Ti. The partial occupation of Si at the Al sites also indicates dependence on the chemical composition and preparation conditions. With a moderate Si content (2 at.%Si), the D0_22_ phase overtakes the D0_23_ phase as the ground phase. This study provides an explanation of the long-standing puzzle that the Al_3_Ti particles observed in most Al-alloys have the D0_22_-type structure,^33–35^ whereas the theoretical calculations predicted that the D0_23_-Al_3_Ti is the ground state in the Al-Ti system.^27,30^

#### Si Solution in Al_3_V

**Fig. 4:**
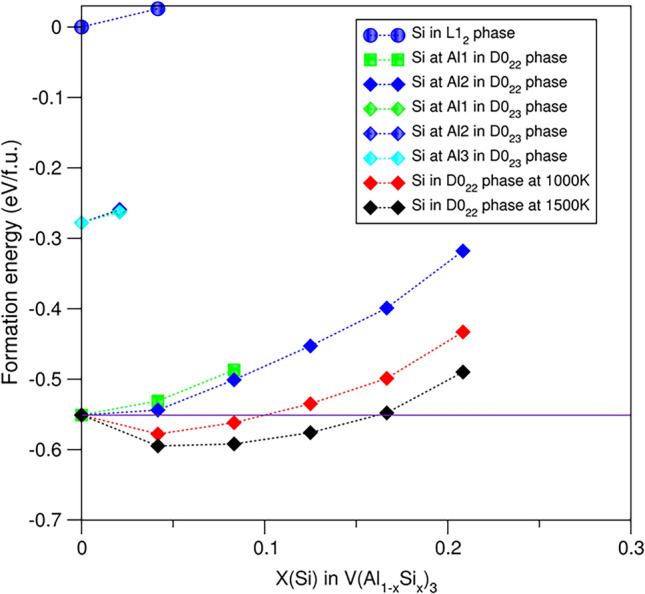
Relationships between the calculated formation energies and Si contents for the Al_3_V phases. Clearly, Si solution in the phases is not favoured with moderate costs for Si solution in D0_22_-Al_3_V. The Si solution occurs at elevated temperatures due to thermodynamic contribution

Figure 4 shows the calculated relationships between the formation energies and Si contents at the Al sites in the Al_3_V phases. There are rather simple relations between the formation energies and Si solution at the Al sites for the Al_3_V phases that it costs energies for Si solution in any of the phases. Thus, there is no change of the phase relations due to Si solution (Fig. 4) and the D0_22_ phase remains the ground state.

Interestingly, the formation energies for Si solution at the Al1 sites are lower than the corresponding one at the Al2 sites in D0_22_-Al_3_V. This is opposite to the cases in D0_22_-Al_3_Sc and Al_3_Ti.

Figure 4 shows that for D0_22_-Al_3_V the costs for Si solution in this phase are moderate. It is reasonable to consider its stability at elevated temperatures. Configurational entropy contributions were estimated for Si solutions at the Al sites in D0_22_-Al_3_V using the same approach as in subsection "Si Solution Stabilizes D0_22_-Al_3_Ti." The obtained data for the Gibbs energies at 1000 K (about the casting temperature) and 1500 K (around the formation temperature of Al_3_V phase^6^) were plotted into Fig. 4.

The Si regions of negative Gibbs energy of the system are about 11 at.% at 1000 K and 17 at.% at 1500 K, respectively. Therefore, D0_22_-(Al, Si)_3_V may be formed and stable at elevated temperatures.

Overall, Si solution in Al_3_V is not favoured. Meanwhile, energy costs for Si solution in D0_22_-(Al,Si)_3_V are moderate, indicating occurrence of Si solution at high temperatures. Naturally, the Si content in a prepared sample depends on the chemical composition and experimental conditions.

### Crystal Chemistry of the (Al_1−***x***_Si_***x***_)_3_T Phases

#### Structural Properties of the Highly Stable (Si-doped) Al_3_T Phases

**Fig. 5:**
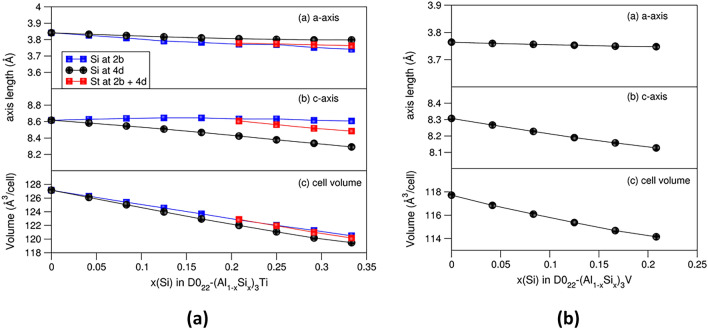
Dependences of lattice parameters and cell volumes on Si content at the Al1 and Al2 sites in D0_22_-(Al_1−*x*_Si_*x*_)_3_Ti (a) and at the Al2 sites in D0_22_-(Al_1−*x*_Si_*x*_)_3_ V (b). Clearly lengths of the lattice parameters and the cell volumes decrease with increasing Si content

The present first-principles calculations showed a rich variety of Si stabilization effects in Al_3_T phases. The binary cubic L1_2_-Al_3_Sc is the ground state, and Si solution in this phase is energetically costly. Thus, Si content in L1_2_-Al_3_Sc is moderate even at elevated temperatures. Meanwhile, Si solution at the Al sites in D0_22_-Al_3_Ti is favoured. Si solution in D0_22_-Al_3_V costs only moderate energy and thus occurs at elevated temperatures. Therefore, it is likely to obtain D0_22_-(Al_1−*x*_Si_*x*_)_3_V samples via, e.g., quenching approaches. The influences of Si content in the D0_22_-(Al_1−*x*_Si_*x*_)_3_T (T = Ti, V) on their crystal structures become important for characterizing the phase in Al alloys. The relationships between the lattice parameters and Si contents for the D0_22_-Al_3_T (T = Ti and V) are presented in Fig. 5.

As shown in Fig. 5, the lattice parameters and corresponding volumes for both D0_22_-Al_3_T (T = Ti and V) systems decrease with increasing Si content in common. This general trend is in line with the smaller atomic radius for Si (1.15 Å) than that of Al (1.43 Å)^53^ and with the calculations that the shorter Al-Si bonds for the dilute Si solute in Al (1.84 Å) than that of the Al-Al bonds (1.86 Å) (Table I).

Figure 5a shows that for D0_22_-(Al_1−*x*_Si_*x*_)_3_Ti, the length of the *a*-axis for the configurations with Si at Al2 decreases more rapidly than that for those with Si at the Al1 (2b) sites. Opposite behaviour was uncovered for the *c*-axis whose length decreases more quickly for Si at the Al2 (4d) sites. The volume of the configurations with Si at Al1 decreases more quickly as well.

The lattice parameters for the most stable configuration, D0_22_-(Al_0.833_Si_0.167_)_3_Ti with Si at the Al1 (Wyckoff 2b) sites, are *a* = 3.810 Å and *c* = 8.468 Å, which are about 5.2% and 3.8%, respectively, smaller than those of D0_22_-Al_3_Ti (Table II). Figure 3 shows that at 1000 K the Gibbs energies are moderate in the range of Si contents (*x*) from 0.125 to 0.333, in which the *a*-/c-axes have lengths between 3.79 Å/8.64 Å and 3.74 Å/8.61 Å for Si at the Al1 sites and 3.82 Å/8.52 Å and 3.80 Å/8.2 Å for Si at the Al2 sites (Fig. 5), respectively. This helps understand the rich variation of the lattice parameters from the experiments.^27,33–35^

Si solution in D0_22_-Al_3_V is possible at elevated temperatures (Fig. 4). The lattice parameters (*a*/*c*) decrease moderately from 3.764 Å/8.307 Å (*x*(Si) = 0) to 3.756 Å/8.227 Å (*x*(Si) = 8.3%). The observed large range of the lattice parameters (Table II) in the experiments^36,51^ may come from a different origin.

#### Electronic Properties of and Chemical Bonding in the Stable (Al_1−***x***_Si_x_)_3_T Phases

**Fig. 6:**
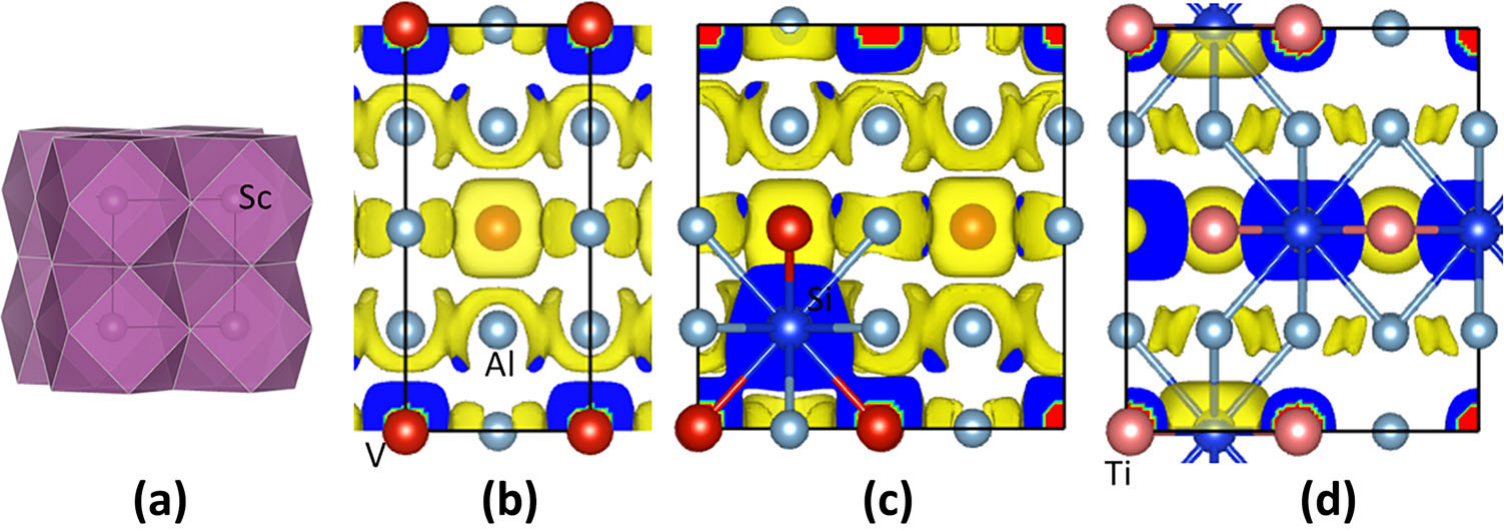
Schematic coordination and electron density distributions in L1_2_-Al_3_Sc (a), D0_22_-Al_3_V (b), D0_22_-(Al_0.958_Si_0.042_)_3_V (c) and D0_22_-(Al_0.833_Si_0.167_)_3_Ti (d) along (approximately for a) the [100] orientation. The black lines represent the *b*-axis (horizontal) and *c*-axis (vertical). The bonds between Si and T/Al are shown for the Si-doped systems (c) and (d)

**Fig. 7:**
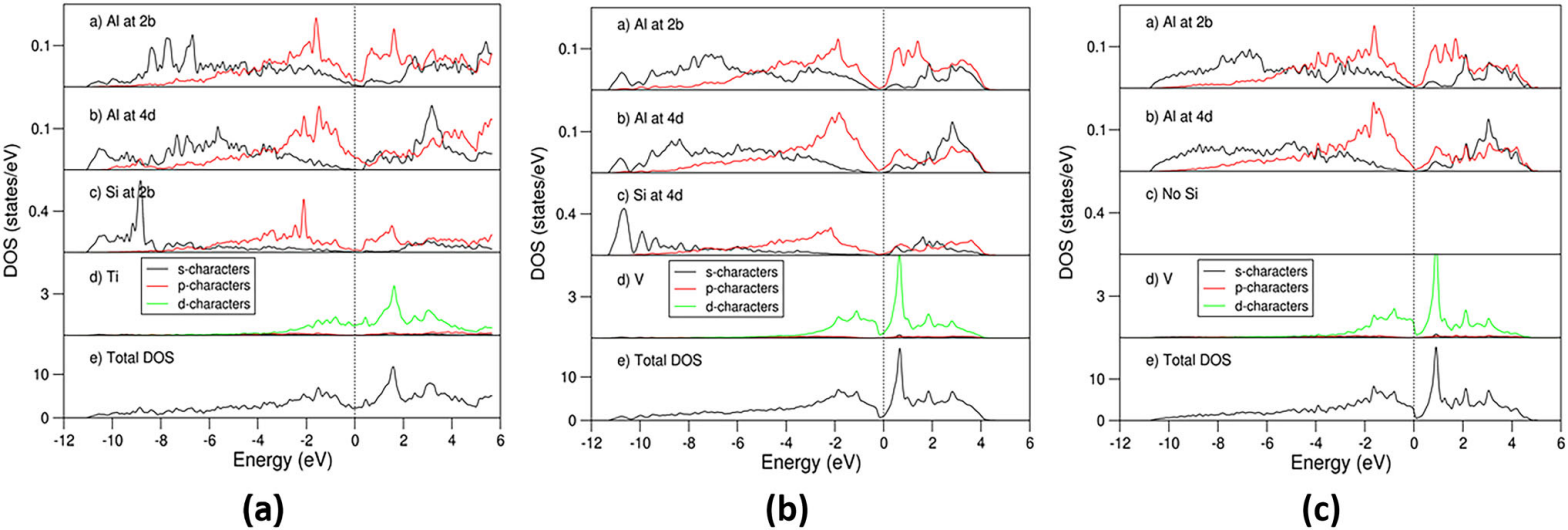
Partial density of states of selected atoms (pDOS) in and total density of states (tDOS) of the highly stable D0_22_-(Al_0.833_Si_0.167_)_3_Ti (a), D0_22_-Al_3_V (c) and D0_22_-(Al_0.958_Si_0.042_)_3_V (b). The unit is states/eV per atom for the pDOS curves and states/eV per primitive unit cell for the tDOS curves. The vertical dotted lines at zero eV represent the Fermi level

**Fig. 8:**
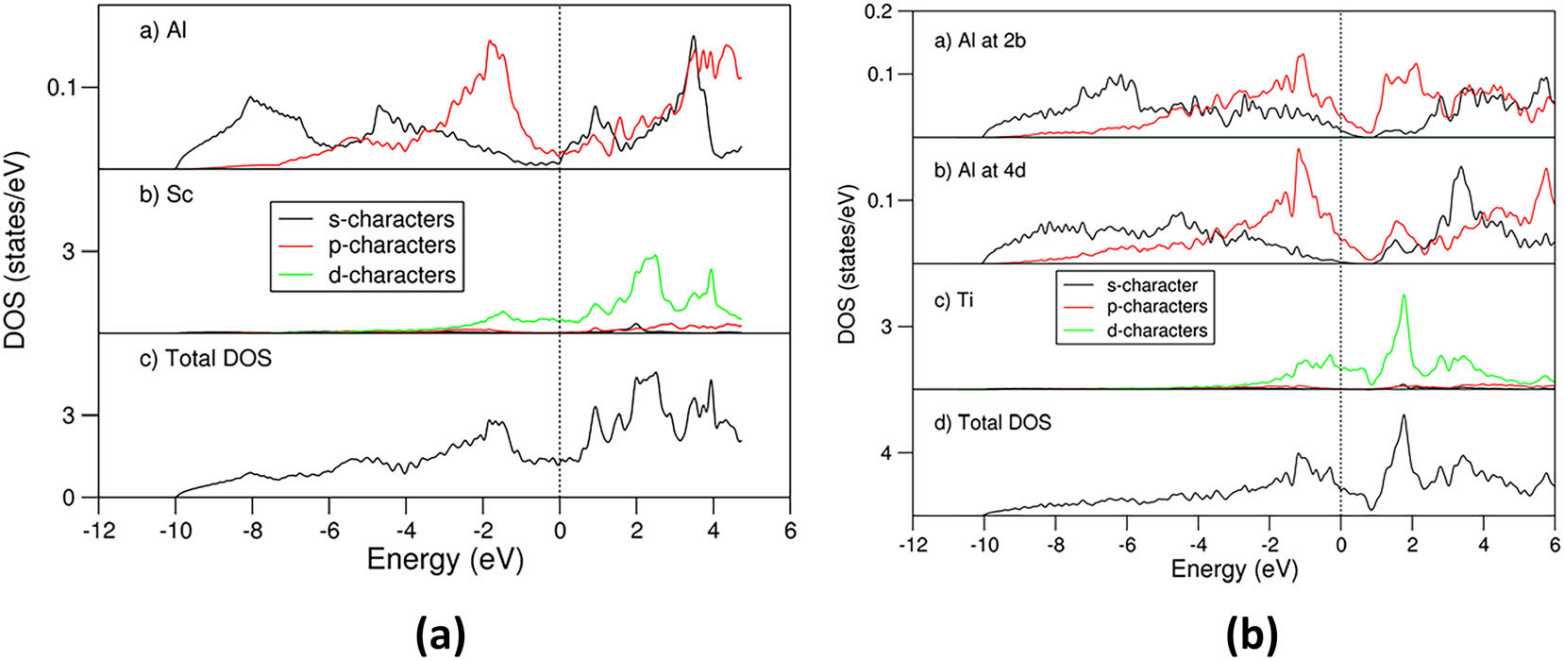
pDOS in and tDOS of L1_2_-Al_3_Sc (a) and novel D0_22_-Al_3_Ti (b). The unit is states/eV per atom for the pDOS curves and states/eV per primitive unit cell for the tDOS curves. The vertical dotted lines at zero eV represent the Fermi level

Electronic band structure calculations were worked out for the highly stable L1_2_-Al_3_Sc, D0_22_-(Al_0.833_Si_0.167_)_3_Ti and D0_22_-Al_3_V and meta-stable D0_22_-(Al_0.958_Si_0.042_)_3_V. The obtained local coordination of Sc by Al atoms in the L1_2_-Al_3_Sc is shown in Fig. 6a, while the electron density distributions in the rest of the above-mentioned phases are shown in Fig. 6b–d. The curves of the related densities of states for D0_22_-(Al_0.833_Si_0.167_)_3_Ti and D0_22_-Al_3_V and D0_22_-(Al_0.958_Si_0.042_)_3_V are shown in Fig. 7, whereas the DOS curves for L1_2_-Al_3_Sc and novel D0_22_-Al_3_Ti are shown in Fig. 8a, b, respectively, with the latter for comparison.

The cuboctahedral coordination of Sc by Al in L1_2_-Al_3_Sc is shown clearly in Fig. 6a. Symmetries and the overlapping between the electron densities in the Al-V-Al chains in D0_22_-Al_3_V in the *a*-*b* plane are presented in Fig. 6b. Figure 6c also shows the overlapping of electron clouds of Si with those of V, indicating local strong Si-V bonding. For D0_22_-(Al_0.833_Ti_0.167_)_3_Ti, there is electron overlapping in the Ti-Si-Ti chains parallel to the *b*-axis, indicating chemical bonding between Ti and Si (Fig. 6d).

The curves of the calculated partial density of states for the Al and Sc atoms in and the total density of states of L1_2_-Al_3_Sc are shown in Fig. 8a. The present calculated results agree with the previous calculations by Duan et al. who employed the first-principles density functional theory within the local-density approximation.^54^ The Al 3 s states dominate the lower part of the valance band (− 10.0 eV to − 3.0 eV) (Fig. 8a) while the DOS curve around the Fermi level is dominated by Sc 3d states mixed with Al 3p states (from − 3.0 eV to the Fermi level, zero eV). There is a broad valley ranging from − 0.9 eV to + 0.5 eV in which the Fermi level falls. Such low DOS at the Fermi level indicates electronic stability of this crystal according to Stoner’s theory.^55^ This agrees with the energy cost for Si solution.

Figure 7c shows the DOS curves for D0_22_-Al_3_V. The DOS curves can be divided into a valence band (from − 10.7 eV to 0.0 eV) and the conduction band above the Fermi level above with a pseudo-gap in between. The lower part of the valence band (from the bottom to − 3.0 eV) is dominated by Al 3 s states while the upper part (− 3.0 eV to 0.0 eV) is dominated by V 3d and Al 3p states. The Fermi level is just at the start of the valley, which is determined by the V 3d states.

The overall shapes of the DOS curves for the Si doped, D0_22_-(Al_0.958_Si_0.042_)_3_V (Fig. 7b) are similar to the corresponding binary Al_3_V (Fig. 7c). A close look reveals differences. First addition of Si causes lowering of the valence band to − 11.3 eV. This is due to mixing of the Si 3 s states which dominated the lower part of the valence band from − 11.3 eV to − 8.0 eV with the Al 3 s states. Second, the Fermi level shifts from the start of the DOS valley to its middle (Fig. 7b) from the beginning of the DOS curve in the parent binary (Fig. 7c). The higher DOS at the parent D0_22_-Al_3_V (Fig. 7c) might be the cause of moderate Si solution at the Al sites, which may shift the Fermi level to the middle of the DOS valley dominated by V 3d states to enhance the stability of the compound.

The overall shapes of the DOS curves of D0_22_-(Al_0.833_Si_0.167_)_3_Ti (Fig. 7a) are also similar to the corresponding ones of D0_22_-(Al_0.958_Si_0.042_)_3_V (Fig. 7b). Meanwhile, some subtle differences can be recognized: (1) the Si 3 s states show more localization between the bottom to − 8.0 eV in the valence band in the former than those in the latter; (2) the sum of the occupied Ti 3d states in Fig 7a is notably smaller than that in Fig. 7b, indicating more 3d electrons in V and in Ti. The tDOS curve around the Fermi level in D0_22_-(Al_0.833_Si_0.167_)_3_Ti is dominated by Ti 3d state and forms a plateau with electron density of states at Fermi level, being 0.71 states/eV for Ti 3d states. In comparison, the total DOS curve of the binary D0_22_-Al_3_Ti has a deep valley at 0.86 eV (see Fig. 8b). Using rigid band filling model, we may conclude that replacement of Al by Si adds electrons into the system and shifts the Fermi level at a high density of the binary compound (Fig. 8b) to the DOS valley at a higher energy (Fig. 7a), which enhances the stability of the system according to the criteria.^55^ This is the physics behind the stability effect of Si solution in D0_22_-Al_3_Ti.

### Electron Count and the (Al_1−*x*_Si_*x*_)_3_ T Phases

The early 3d transition metals belong to the s-d elements with electronic configurations: Sc [Ar] 3d^1^ 4s^2^, Ti [Ar] 3d^2^ 4s^2^ and V [Ar] 3d^3^ 4s^2^; here [Ar] represents the close-shell core electrons. Our calculations showed a trend between the electron number and the energetic hierarchy of the phases: by increasing electron number, the preferred structure is L1_2_ for ScAl_3_ to D0_23_ for TiAl_3_ and to D0_22_ for VAl_3_. Analogous behaviour is expected for the related TAl_3_ compounds with T = the early 4d/5d transition metals (Y/Lu, Zr/Hf and Nb/Ta).^39,43,52,56^ Such an energetic hierarchy and phase relations have been confirmed by our systematic first-principles calculations for the TAl_3_ family (Table III) with the same code and settings described in section "Details of Computations."

**Table III:** Calculated formation energies (Δ*E*_*1*_) for the TAl_3_ (T = Sc/Y/La, Ti/Zr/Hf, V/Nb/Ta) phases and related electronic configurations of the early *n*d (*n* = 3, 4, 5) transition metals according to Eq. 1

Phase	Formation energy Δ*E*_*1*_ (eV/f.u.) and related electronic configurations of T								
	Al_3_Sc (3d^1^4s^2^)	Al_3_Ti (3d^2^4s^2^)	Al_3_V (3d^3^4s^2^)	Al_3_Y (4d^1^5s^2^)	Al_3_Zr (4d^2^5s^2^)	Al_3_Nb (4d^3^5s^2^)	Al_3_La (5d^1^6s^2^)	Al_3_Hf (5d^2^6s^2^)	Al_3_Ta (5d^2^6s^2^)
L1_2_	**0.00**	0.00	0.00	**0.00**	0.00	0.00	**0.00**	0.00	0.00
D0_22_	0.36	−0.11	**−0.54**	0.51	0.00	**−0.66**	0.59	−0.08	**−0.67**
D0_23_	0.18	−**0.13**	-0.27	0.26	**−0.10**	−0.33	0.28	**−0.11**	−0.34

The most stable phases are marked in bold.

Table III shows clearly the relation between the preferred structure and electron count in the TAl_3_ family. With increasing number of electrons, the preferred structure transits from L1_2_- for T = Sc/Y/La (d^1^ elements) to D0_23_ for T = Ti/Zr/Hf (d^2^) and further to D0_22_ for T = V/Nb/Ta (d^3^).

Si replacing Al in TAl_3_ indicates an increasing number of electrons in the system. Correspondingly, it may cause phase changes. This is shown by Si dissolving at the Al1 sites in D0_22_-ScAl_3_ (Fig. 2). The remarkable stabilization effect is Si dissolving in D0_22_-TiAl_3_. As shown in Fig. 3, a moderate content of dissolving Si causes transition from the D0_23_-TiAl_3_ to D0_22_-Ti(Al_1−*x*_Si_*x*_)_3_ (*x* > 0.02). For D0_22_-VAl_3_, Si dissolving has little stabilization effect, since it already contains the highest number of electrons for this family. Naturally, this stabilization effect due to an increasing number of electrons holds for the related TAl_3_ with T = Y/La, Zr/Hf and Nb/Ta.

Table III also shows notable energetic differences for the TAl_3_ phases with different transition metals. For example, the energy difference between D0_22_- and D0_23_-TiAl_3_ is about 0.02 eV/f.u., which is notably smaller than that for ZrAl_3_ (0.10 eV/f.u.). Correspondingly, the minimal Si content for the stabilized D0_22_-Ti(Al_1−*x*_Si_*x*_)_3_ is small (*x* ~ 0.02), whereas it will be larger for stabilizing the D0_22_-Zr(Al_1−*x*_Si_*x*_)_3_ over the D0_23_-ZrAl_3._ Moreover, replacing Al by an element with fewer valence electrons, such as alkaline earth or noble metal elements, reduces the electrons in the system, which may change the energetic hierarchy and the phase relation in the TAl_3_ compounds in a reverse way. Effects of impurity solution in the TAl_3_ compounds listed in Table III and for T = , e.g., Cr/Mo/W (d^4^) elements, deserve further investigation.

## Conclusion

First-principles density-function theory calculations for Si solution on the L1_2_-, D0_22_- and D0_23_-Al_3_T (T = Sc, Ti and V) phases showed that Si prefers uniform distribution in the same type of Al site. Si solution has a rich variety of effects on the stability of the Al_3_T phases.

There is a link between the number of electrons in TAl_3_ and the preferred structures: L1_2_-ScAl_3_, D0_23_-TiAl_3_ and D0_22_-VAl_3_. Si replacing Al increases the number of electrons in the systems, which may cause change of the corresponding preferred structures. This link also holds for the corresponding early 4d and 5d transition metal tri-aluminides.

Si solution stabilizes D0_22_-Al_3_Ti phase so that in the Si partially dissolved phase it goes by the binary D0_23_-Al_3_Ti. The partial substitution also induces extra freedom for the Si content at high temperatures. This explains the widely observed D0_22_-type particles in the commercial Al-based alloys, which contain variable degrees of Si. The most stable configuration has chemical formula D0_22_-(Al_0.833_Si_0.167_)_3_Ti.

Si can be dissolved at the Al1 and Al2 sites and stabilize both D0_22_- and D0_23_-Al_3_Sc phases. However, the Si stabilization is not enough to change their phase relations so that the binary L1_2_-Al_3_Sc structure keeps the ground-state phase. The high energy cost indicates low Si doping in the cubic L1_2_-Al_3_Sc crystals even at elevated temperatures.

Si solution is not favoured in the Al_3_V phases. Meanwhile, the energy cost for Si solution in the D0_22_ phase is moderate. Therefore, configurational entropy contribution enables moderate Si solution in D0_22_-Al_3_V at elevated temperatures.

Crystallographically, Si solution reduces the lattice parameters in an almost linear way. This agrees with the rich variation of the lattice parameters for the D0_22_-(Al,Si)_3_Ti phase observed in the manufactured Al alloys.

The electronic structure calculations showed strong bonding between Si and the transition metals. The Fermi level falls in the DOS valleys for the highly stable L1_2_-Al_3_Sc, D0_22_-(Al_0.833_Si_0.167_)_3_Ti and Si moderately doped D0_22_-(Al_1−*x*_Si_*x*_)_3_V (e.g., *x* ~ 0.04) phases.
